# Transcriptional Landscape of a *bla*_KPC-2_ Plasmid and Response to Imipenem Exposure in *Escherichia coli* TOP10

**DOI:** 10.3389/fmicb.2018.02929

**Published:** 2018-12-03

**Authors:** Agnès B. Jousset, Isabelle Rosinski-Chupin, Julie Takissian, Philippe Glaser, Rémy A. Bonnin, Thierry Naas

**Affiliations:** ^1^Department of Bacteriology-Parasitology-Hygiene, Bicêtre Hospital, Assistance Publique-Hôpitaux de Paris, Le Kremlin-Bicêtre, France; ^2^Associated French National Reference Center for Antibiotic Resistance, Le Kremlin-Bicêtre, France; ^3^EA7361 “Structure, dynamic, function and expression of broad spectrum β-lactamases”, Faculty of Medicine, Paris-Sud University, Le Kremlin-Bicêtre, France; ^4^Joint Research Unit Evolution and Ecology of Resistance to Antibiotics, Institut Pasteur-APHP-University Paris Sud, Paris, France; ^5^CNRS, UMRS 3525, Paris, France

**Keywords:** carbapenemase, KPC-producing plasmids, transcriptome, RNA-seq, oxidative-stress

## Abstract

The diffusion of KPC-2 carbapenemase is closely related to the spread of *Klebsiella pneumoniae* of the clonal-group 258 and linked to IncFII_K_ plasmids. Little is known about the biology of multi-drug resistant plasmids and the reasons of their successful dissemination. Using *E. coli* TOP10 strain harboring a multi-replicon IncFII_K_-IncFIB *bla*_KPC−2_-gene carrying plasmid pBIC1a from *K. pneumoniae* ST-258 clinical isolate BIC-1, we aimed to identify basal gene expression and the effects of imipenem exposure using whole transcriptome approach by RNA sequencing (RNA-Seq). Independently of the antibiotic pressure, most of the plasmid-backbone genes were expressed at low levels. The most expressed pBIC1a genes were involved in antibiotic resistance (*bla*_KPC−2_, *bla*_TEM_ and *aph*(3′)-I), in plasmid replication and conjugation, or associated to mobile elements. After antibiotic exposure, 34% of *E. coli* (pBIC1a) genome was differentially expressed. Induction of oxidative stress response was evidenced, with numerous upregulated genes of the *SoxRS/OxyR* oxydative stress regulons, the Fur regulon (for iron uptake machinery), and *IscR* regulon (for iron sulfur cluster synthesis). Nine genes carried by pBIC1a were up-regulated, including the murein DD-endopeptidase *mepM* and the copper resistance operon. Despite the presence of a carbapenemase, we observed a major impact on *E. coli* (pBIC1a) whole transcriptome after imipenem exposure, but no effect on the level of transcription of antimicrobial resistance genes. We describe adaptive responses of *E. coli* to imipenem-induced stress, and identified plasmid-encoded genes that could be involved in resistance to stressful environments.

## Introduction

*Klebsiella pneumoniae* is a prominent opportunistic pathogen for hospital- and community-acquired infections (Navon-Venezia et al., 2017). The increasing incidence of KPC-producing *K. pneumoniae* (KPC-*Kp*) in health care facilities is a cause of global concern, especially in countries where the carbapenemase KPC is endemic, e.g., the United States, Israel, Greece, and Italy (Bonomo et al., 2017). The *bla*_KPC_ gene, coding for an Ambler class A carbapenemase is mostly associated with *K. pneumoniae* but has been reported to a lesser extent in other Enterobacteriacae, in *Pseudomonas* spp. and in *Acinetobacter baumannii* (Cuzon et al., 2011, 2013). KPC confers resistance or decreased susceptibility to almost all β-lactams, and KPC-producing isolates are often resistant to many other non-β-lactam drugs because of the co-occurrence of *bla*_KPC_ gene with resistance genes to other classes of antibiotics. This multidrug resistance leaves only limited therapeutic options for antimicrobial treatment, and thereby results in high mortality rates (Lee and Burgess, 2012).

The *bla*_KPC_ genes have been identified on several transferable plasmids of different incompatibility goups (e.g., IncFII_K_, IncA/C, IncF, IncN, IncP, IncR, IncX, and ColE1), but IncFII_K_-type plasmids are the most common and are involved in their spreading (Chen et al., 2014b). The pKpQIL plasmid was the first characterized IncFII_K2_ *bla*_KPC_-carrying plasmid in Israel in 2006, and is strongly associated to the *K. pneumoniae* ST258 pandemic clone (Chen et al., 2013). Subsequently, pKpQIL-like plasmids seemed to have played a major role in KPC dissemination with several reports worldwide (Baraniak et al., 2011; García-Fernández et al., 2012; Hidalgo-Grass et al., 2012; Chen et al., 2013, 2014a; Papagiannitsis et al., 2016; Doumith et al., 2017). Complete sequencing of KPC-associated plasmids revealed their plasticity with multiple rearrangements that occurred between pKpQIL-like plasmids and pKPN3, a non-KPC-encoding IncFII_K1_-FIB plasmid described in *K. pneumoniae* MGH78578 (also known as ATCC 700721; Chen et al., 2013; Jousset et al., 2018). However, little is known about their basic biology, and due to the presence of multiple antimicrobial resistance genes on IncFII_K_ plasmids, their broad host range, and their rapid dissemination, there is a need to understand the mechanisms underlying regulation and maintenance of these plasmids.

KPC is mostly associated to *K. pneumoniae* of a single clonal group (CG) designated CG258 containing 43 different MLST single-locus variants with ST258, ST11, ST512, and ST340 being the more predominant ones (Deleo et al., 2014; Rojas et al., 2017, 2018). KPC has also been identified in other Enterobacteriaceae such as *Escherichia coli, Citrobacter* sp, *Enterobacter* sp, *Serratia marcescens, Proteus. Mirabilis*, and *Morganella morganii*. KPC-producing *E. coli* isolates usually have low MICs for carbapenems and can remain susceptible according to clinical breakpoints (Landman et al., 2010). Regardless the level of carbapenem resistance, options for treatment of infections due to KPC-producing Enterobacteriaceae are limited and current clinical evidence for treatment guidelines are still lacking. Even if novel β-lactamase inhibitors (such as avibactam) are increasingly used with success (van Duin et al., 2017), carbapenem-based regimens remain a therapeutic option in many countries. Imipenem and meropenem remain useful mainly in combination with other classes of antibiotics or even another carbapenem, such as ertapenem (Bonomo et al., 2017). Pharmacokinetic parameters of the used molecule and Minimal Inhibitory Concentration value of the KPC-producing strain are also crucial in patient outcome (Tumbarello et al., 2012; Daikos et al., 2014). Tumbarello et al. showed that patients infected by carbapenemase-producing Enterobacteriaceae with imipenem MIC values ≥4 μg/mL had worse outcomes than patients whose isolates had MIC ≤ 2 μg/mL. On the contrary, therapeutic failure with use of carbapenems against KPC-2-producing isolates possessing low MICs were also reported (Weisenberg et al., 2009; Daikos and Markogiannakis, 2011). Previous studies were made to understand the transcriptional regulation of *bla*_KPC_ revealing the versatile expression of this gene (Naas et al., 2012; Cheruvanky et al., 2017; Girlich et al., 2017). Measuring *bla*_KPC_ RNA expression revealed that *bla*_KPC_ gene copy number and the presence of different promoters cannot be strictly correlated with MICs determination and are not sufficient to explain the level of resistance regarding carbapenems (Roth et al., 2011; Naas et al., 2012). These data triggered our interest in the regulation of *bla*_KPC_ gene and in the entire plasmid transcriptome, in particular under antibiotic exposure.

Effect of β-lactams on *E. coli* has been characterized using transcriptomic approaches. In addition to interacting with their targets, bactericidal antibiotics induce parallel changes in bacterial metabolism that promote the formation of reactive oxygen species, which play a role in cell death (Kohanski et al., 2007; Dwyer et al., 2014). β-lactams were also shown by Miller and colleagues to trigger the SOS response *via* the activation of the DpiAB two-component system (Miller, 2004). However, the dynamic underlying oxidative and antibiotic-induced SOS stress response activation needs further exploration. In most of these studies, sub-inhibitory doses of ampicillin were used on fully susceptible *E. coli* isolates expressing no β-lactamase.

To give new insights into the biology of multidrug resistance successful plasmids, the transcriptome of *E. coli* harboring pBIC1a, an IncFII_K2_-FIB-type *bla*_KPC−2_ carrying plasmid was performed. To gain more information about the complex regulation of the *bla*_KPC_ gene and all the transcripts of the cell, the transcriptome of *E. coli* harboring pBIC1a was performed with or without imipenem exposure. Moreover, to mimic physiopathological conditions that occur during a carbapenem-based treatment, we chose to analyse the response of *E. coli* (pBIC1a) to high doses of imipenem (10 times the MIC value of the strain). We described adaptive responses of *E. coli* (pBIC1a) to imipenem-induced stress with disruption of several metabolic pathways.

## Results and discussion

### Mosaic structure of pBIC1a plasmid

**Figure 1:**
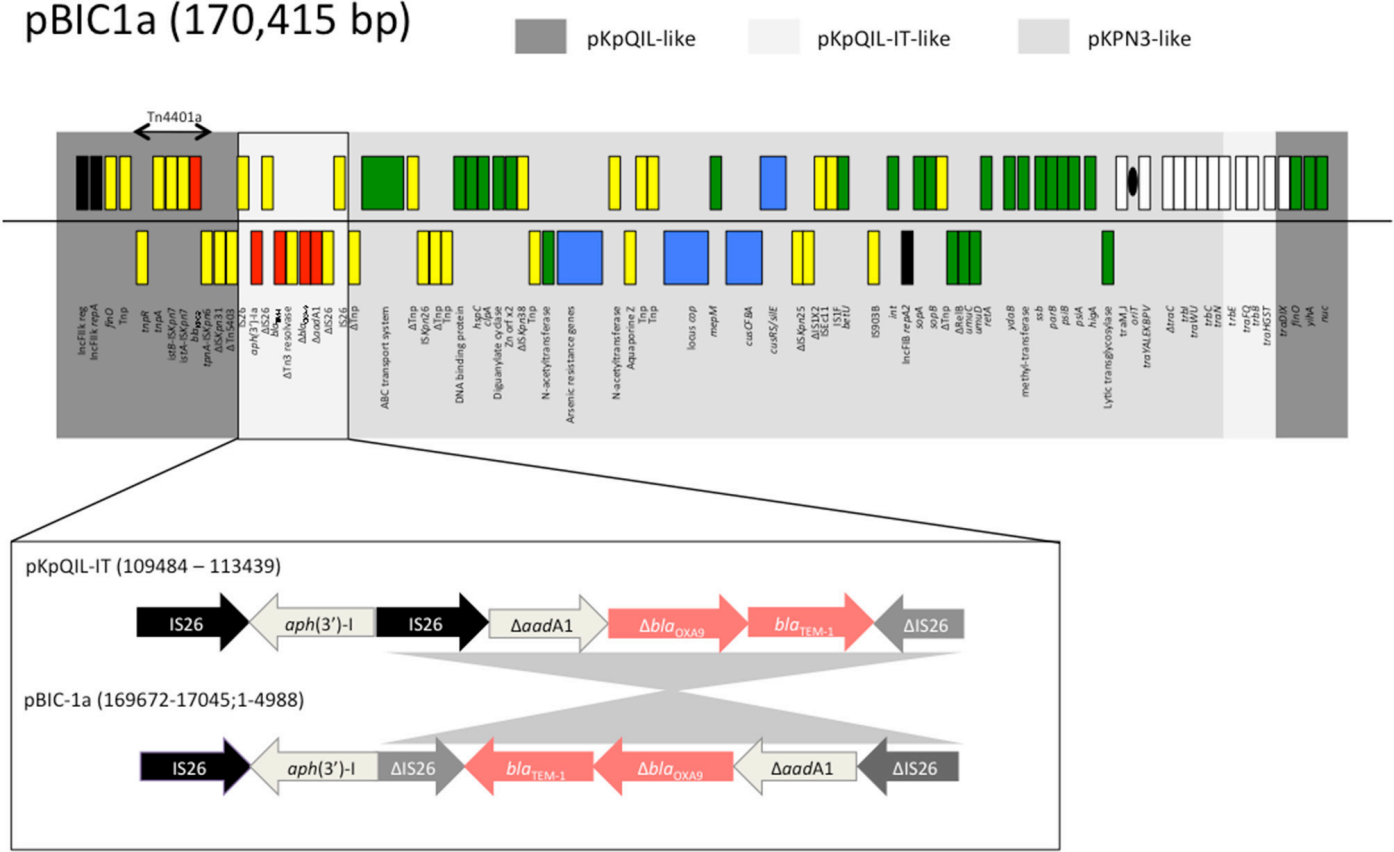
Schematic representation of plasmid pBIC-1a main features. Gray boxes indicate regions with high nucleotide identity (>97%) with pKPN3 (NC_009649.1), pKpQIL (GU595196.1), and pKpQIL-IT (NC_019155.1) plasmids. Genes from the forward strand are represented above the line, and genes from the reverse strand below. Antimicrobial resistance genes are indicated in red, the replicase genes in black, the heavy metal resistance operons in blue, the transfer operon in white, the genes involved in genetic mobile elements in yellow, and other plasmid-located genes in green. Δ indicates a truncated gene. *Tnp*: transposase. As for pKpQIL-IT, pBIC-1a carries an aminoglycoside resistance gene on a putative composite transposon-like element, IS*26*-*aph*(3′)-Ia-ΔIS*26*, located downstream of Tn*4401a*. Unlike in pKpQIL-IT, the IS*26* located downstream of *aph*(3′)-Ia is truncated. In addition, an inversion of 3,872 nt fragment is observed as compared to pKpQIL-IT.

Plasmid pBIC1a is a *bla*_KPC−2_-encoding plasmid of 170,415 bp in-size from a ST258 *K. pneumoniae* BIC-1 (Jousset et al., 2018). This isolate was recovered from a contaminated endoscope in France in 2009 (Naas et al., 2010). This plasmid exhibited an average GC content of 53% and 193 predicted open reading frames (ORF). The overall structure of pBIC1a was highly similar (83% query coverage and 99% nucleotide identity by Blast) to the non-KPC carrying plasmid pKPN3 (Figure 1). Plasmids pBIC1a and pKPN3 shares a common region of 120-kb. This region contains genes responsible for plasmid replication, maintenance, transmission, and heavy metal (arsenic, copper, and silver) resistance, and presents the replicase *repA2* of IncFIB type.

The rest of pBIC1a plasmid shared homology with pKpQIL-like plasmids such as pKpQIL (27% query coverage and 99.9% nucleotide identity) and pKpQIL-IT (36% query coverage and 99.3% nucleotide identity; Figure 1). This region contains the end of the *tra* operon, another replicase *repA* of IncFII_K2_ type according to replicon sequence typing (Carattoli et al., 2014), making pBIC1a a multi-replicon IncFII_K2_-IncFIB plasmid. The multi-replicon status can expand plasmid's host range replication (Villa et al., 2010). Like most of the pKpQIL-like plasmids, the variant *bla*_KPC−2_ gene was found as part of the class II transposon Tn*4401a*. Additional antimicrobial resistance genes were present: *bla*_TEM−1_, *bla*_OXA−9_ (disrupted by a frameshift mutation), *aph(3*′*)-I* and partial *aadA1*. Furthermore, when compared to original pKpQIL, pBIC1a had an additional truncated resistance gene carried on a composite transposon-like element, IS*26*-Δ*aphA1*-ΔIS*26*, located downstream of Tn*4401a*. This element has been described in pKpQIL-IT, but differed by an inversion of a 3,872 nt fragment that occurred downstream (Figure 1).

**Figure 2:**
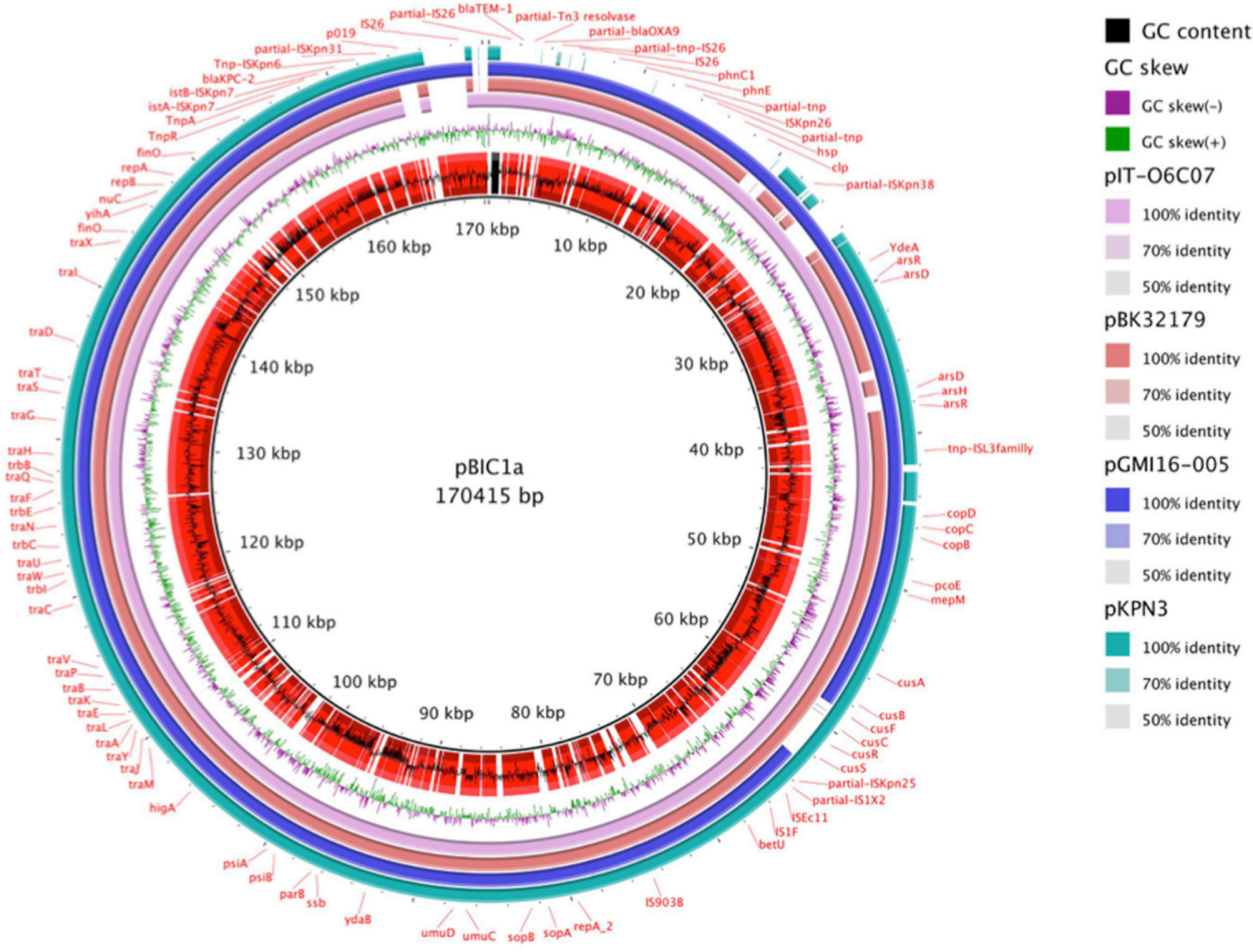
Nucleotidic alignment of *bla*_KPC_-carrying plasmids deriving from pKPN3. pKPN3 (NC_009649.1) is a plasmid from *K. pneumoniae* MGH78578 that carries no β-lactamase. pIT-O6C07 (LT009688.1), pBK32179 (JX430448), pGMI16-005_01 (NZ_CP028181.1) are KPC-producing plasmids deriving from recombination between pKPN3 and pKpQIL-like plasmids. From the inside to the outside, the different circles represent: pBIC1a GC content (black) and open reading frames annotation (red), pBIC1a GC skew (purple and green), alignment with pIT-O6C07 (pink), alignment with pBK32179 (red), alignement with pGMI16-005 (blue), alignment with pKPN3 (green). This circular representation was performed using BRIG.

Therefore, pBIC1a seems to be the result of several transpositions and recombination events between pKpQIL, pKpQIL-IT, and pKPN3 plasmids (Figure 2). Differently rearranged pKPN3 plasmids have been described in KPC-*Kp* from the USA, Greece and Italy (Chen et al., 2013; Wright et al., 2014; Papagiannitsis et al., 2016; Doumith et al., 2017). pIT-O6C07 (LT009688.1), pBK32179 (JX430448) and pGMI16-005_01 (NZ_CP028181.1) are three fully sequenced IncFII_K2_-IncFIB plasmids carrying *bla*_KPC_, resulting from recombination between pKPN3 and pKpQIL-like plasmids (Figure 2). pIT-O6C07 is the closest to pBIC1a with 97% query coverage and 99% nucleotide identity (Papagiannitsis et al., 2016). The two plasmids only differed by a 4,695 nt region containing *bla*_TEM−1_ and *bla*_OXA−9_, that is missing in pIT-O6C07. Alignment of the four KPC-producing plasmids deriving from pKPN3 indicated that recombination did not occur at the same spot precisely (Figure 2). Recombination events between pKpQIL-like and pKPN3-like plasmids seem common, likely due to the presence of highly homologous regions and their frequent coexistence in *K. pneumoniae*.

### Transcriptome analysis of *E. coli* (pBIC1a)

*E. coli* TOP10, transformed with the KPC-carrying plasmid pBIC1a, was grown at mid-log phase in rich media (Brain Heart Infusion (BHI) liquid media). Two conditions in biological triplicates were studied: liquid exponential growth as control and liquid exponential growth supplemented with imipenem at final concentration of 5 μg/ml (10 times the MIC value of TOP10-pBIC1a for imipenem, which was determined at 0.5 μg/ml using E-Test). This concentration was chosen because it can be achieved in human serum during the treatment of a bacteriemia due to a KPC-producing Enterobacteriaceae after intravenous administration of 1,000 mg of imipenem-cilastatin (Singlas, 1990; Signs et al., 1992). Imipenem exposure was limited to 10 min to evaluate the immediate cellular response and limit bacterial cell death. The viability of bacterial cells was verified by time-kill analysis and no effect on survival was observed after 10 min exposure to imipenem (Figure S1).

After 10 min of exposure, extraction of total RNA was performed on each replicate. Libraries were obtained from ribo-depleted RNA. After sequencing, a mean of 27.9 ± 3.3 million reads per individual library was obtained. Reads were aligned on *E. coli* TOP10 (CP000948.1) and pBIC1a (CP022574.1) DNA sequences, with 97.25% ± 0.78% of the reads mapping on non-ribosomal regions (Table S1). The principal component analysis (PCA) and the clustering heatmap plots revealed that the samples clustered to their biological replicates (Figure S2).

### Transcriptional landscape of pBIC1a in the absence of imipenem

In BHI medium and exponential growth phase, the replicases *repA* IncFII_K_, *repA* IncFIB, and the regulator *repB* IncFII_K_ genes were expressed with reads per kilobase per million mapped reads (RPKM) values of 127, 224, and 643 respectively. These genes were used for subsequent comparisons as they are involved in plasmid replication and should be expressed consistently during exponential growth due to continuous cell division (Lang et al., 2012).

Alignment of reads on pBIC1a sequence was performed and heatmap visualization was used to identify highly or weakly transcribed regions using IGV software (Robinson et al., 2011).

**Figure 3:**
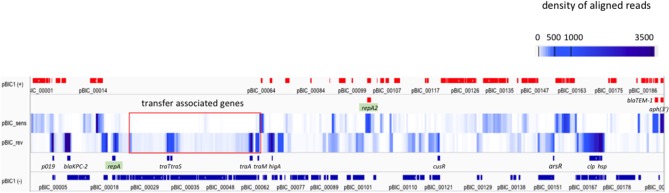
Expression of pBIC1a genes in the absence of antibiotic. The coverage (expressed as the average density of reads over 500 nt fragments) was computed on each strand of pBIC1a sequence and visualized by using a heatmap representation (IGV software). Open Reading Frames (ORFs) annotated on the strand (+ or sens) are indicated in red boxes and ORFs annotated on the strand (– or rev) are indicated in blue boxes. Known annotation of the most expressed genes are mentioned on a supplementary line for both strands. Both replicases (*repA* IncFII_K_-type and *repA2* IncFIB-type) are highlighted in green. Genes associated to plasmid transfer are boxed in red.

Most of the plasmid genes were expressed at low levels or not expressed during exponential phase in BHI medium, with only 34 of predicted CDS (17%) having RKPM values above the *repA* IncFII_K_ gene RPKM value (Table 1; Figure 3). These results are comparable with those found in a previous transcriptomic analysis of a multidrug resistance-encoding plasmid where most of the backbone was transcriptionally silent in *E. coli* (Lang et al., 2012). Among the plasmid scaffold genes, *higA* antitoxin gene, part of HigA/HigB type II toxin/antitoxin system was highly expressed suggesting a role in pBIC1a maintenance in *E. coli*. Most genes involved in plasmid transfer function were expressed at low levels, except the genes encoding TraM (relaxosome protein), TraA (pilin precursor), TraL (pilus assembly protein), TraT (surface exclusion protein), and TraS (entry exclusion protein; Figure 3).

**Table 1:** Expression analysis of pBIC1a genes without imipenem exposure using RPKM values.

**pBIC1a**	**Gene name**	**Orientation**	**Annotation**	**RPKM value**
pBIC_00001		+	Aldehyde dehydrogenase	35
pBIC_00002		+	Putative resolvase	12
pBIC_00003		+	Hypothetical protein	23
pBIC_00004		+	Hypothetical protein	144
pBIC_00005		–	p019 of ISKpn31	1,840
pBIC_00006		–	Transposase	20
pBIC_00007		+	Mobile element protein	8
pBIC_00008		+	Tnp-ISKpn6	8
pBIC_00009	*bla*_KPC−2_	–	KPC-2	2,706
pBIC_00010		–	Hypothetical protein	294
pBIC_00011	*istA*	–	ISKpn7	0
pBIC_00012	*istB*	–	ISKpn7	1
pBIC_00013	*TnpA*	–	Transposase	4
pBIC_00014	*TnpR*	+	Resolvase	13
pBIC_00015		+	Hypothetical protein	2,261
pBIC_00016		+	Hypothetical protein	76
pBIC_00017		+	Transposase	65
pBIC_00018		–	Hypothetical protein	220
pBIC_00019	*finO*	–	IncF plasmid conjugative transfer fertility inhibition protein FinO	328
pBIC_00020	*repA*	–	DNA replication protein (IncFIIk)	127
pBIC_00021	*repB*	–	Replication regulatory protein	643
pBIC_00022	*nucC*	–	Endonuclease	13
pBIC_00023		–	Hypothetical protein	35
pBIC_00024	*yihA*	–	Hypothetical protein	13
pBIC_00025		–	Hypothetical protein	14
pBIC_00026		–	Hypothetical protein	11
pBIC_00027	*finO*	–	IncF plasmid conjugative transfer fertility inhibition protein FinO	72
pBIC_00028	*traX*	–	IncF plasmid conjugative transfer pilin acetylase TraX	14
pBIC_00029	*traI*	–	IncF plasmid conjugative transfer DNA-nicking and unwinding protein TraI	10
pBIC_00030	*traD*	–	IncF plasmid conjugative transfer protein TraD	7
pBIC_00031		–	Hypothetical protein	8
pBIC_00032	*traT*	–	IncF plasmid conjugative transfer surface exclusion protein TraT	282
pBIC_00033	*traS*	–	IncF plasmid conjugative transfer surface exclusion protein TraS	231
pBIC_00034	*traG*	–	IncF plasmid conjugative transfer protein TraG	56
pBIC_00035	*traH*	–	IncF plasmid conjugative transfer pilus assembly protein TraH	6
pBIC_00036		–	Hypothetical protein	4
pBIC_00037	*trbB*	–	IncF plasmid conjugative transfer protein TrbB	7
pBIC_00038	*traQ*	–	IncF plasmid conjugative transfer protein TraQ	6
pBIC_00039	*traF*	–	IncF plasmid conjugative transfer pilus assembly protein TraF	6
pBIC_00040		–	Hypothetical protein	19
pBIC_00041		–	Hypothetical protein	14
pBIC_00042	*trbE*	–	IncF plasmid conjugative transfer protein TrbE	5
pBIC_00043	*traN*	–	IncF plasmid conjugative transfer protein TraN	10
pBIC_00044	*trbC*	–	IncF plasmid conjugative transfer protein TrbC	7
pBIC_00045	*traU*	–	IncF plasmid conjugative transfer pilus assembly protein TraU	10
pBIC_00046	*traW*	–	IncF plasmid conjugative transfer pilus assembly protein TraW	8
pBIC_00047	*trbI*	–	IncF plasmid conjugative transfer protein TrbI	9
pBIC_00048	*traC*	–	IncF plasmid conjugative transfer pilus assembly protein TraC	11
pBIC_00049		–	Hypothetical protein	12
pBIC_00050		–	Hypothetical protein	11
pBIC_00051		–	Hypothetical protein	9
pBIC_00052		–	Hypothetical protein	7
pBIC_00053		–	Hypothetical protein	14
pBIC_00054	*traV*	–	IncF plasmid conjugative transfer pilus assembly protein TraV	22
pBIC_00055	*traP*	–	IncF plasmid conjugative transfer protein TraP	22
pBIC_00056	*traB*	–	IncF plasmid conjugative transfer pilus assembly protein TraB	21
pBIC_00057	*traK*	–	IncF plasmid conjugative transfer pilus assembly protein TraK	25
pBIC_00058	*traE*	–	IncF plasmid conjugative transfer pilus assembly protein TraE	36
pBIC_00059	*traL*	–	IncF plasmid conjugative transfer pilus assembly protein TraL	433
pBIC_00060	*traA*	–	IncF plasmid conjugative transfer pilin protein TraA	656
pBIC_00061	*traY*	–	IncF plasmid conjugative transfer regulator TraY	65
pBIC_00062	*traJ*	–	IncF plasmid conjugative transfer regulator TraJ	34
pBIC_00063	*traM*	–	IncF plasmid conjugative transfer mating signal transduction protein TraM	429
pBIC_00064		+	Lytic transglycosylase	295
pBIC_00065		–	Hypothetical protein	16
pBIC_00066		–	Unnamed protein product	20
pBIC_00067		+	Hypothetical protein	5
pBIC_00068	*higA*	–	Antitoxine HigA	223
pBIC_00069		–	Hypothetical protein phage-related	400
pBIC_00070		–	Hypothetical protein	17
pBIC_00071		–	Hypothetical protein	14
pBIC_00072		–	Hypothetical protein	23
pBIC_00073		–	Hypothetical protein	163
pBIC_00074		–	Hypothetical protein	144
pBIC_00075		–	Hypothetical protein	15
pBIC_00076		+	Mobile element protein	22
pBIC_00077		–	Hypothetical protein	19
pBIC_00078		–	Hypothetical protein	2
pBIC_00079	*psiA*	–	PsiA protein	2
pBIC_00080	*psiB*	–	PsiB protein	3
pBIC_00081		–	Hypothetical protein	4
pBIC_00082	*parB*	–	ParB	5
pBIC_00083	*ssb*	–	Single-stranded DNA-binding protein	7
pBIC_00084		+	Hypothetical protein	5
pBIC_00085		–	SAM-dependent methyltransferase	3
pBIC_00086		–	Hypothetical protein	3
pBIC_00087	*ydaB*	–	YdaB	2
pBIC_00088		–	Hypothetical protein	11
pBIC_00089		–	Hypothetical protein	53
pBIC_00090		–	DNA polymerase III theta subunit	52
pBIC_00091		–	Antirestriction protein klcA	6
pBIC_00092		+	Hypothetical protein	9
pBIC_00093		–	Hypothetical protein	47
pBIC_00094		–	Retron-type RNA-directed DNA polymerase; Ontology_term	49
pBIC_00095	*umuD*	+	Error-prone repair protein UmuD	17
pBIC_00096	*umuC*	+	Error-prone lesion bypass DNA polymerase V (UmuC)	11
pBIC_00097		–	Mobile element protein	15
pBIC_00098		–	Mobile element protein	26
pBIC_00099		+	Hypothetical protein	2
pBIC_00100		–	Hypothetical protein	13
pBIC_00101	*sopB/parB*	–	Chromosome (plasmid) partitioning protein ParB	55
pBIC_00102	*sopA/parA*	–	Chromosome (plasmid) partitioning protein ParA	53
pBIC_00103	*repA2*	+	Replicase (IncFI)	224
pBIC_00104		–	Resolvase/Recombinase	36
pBIC_00105		+	Hypothetical protein	3
pBIC_00106		+	Sensor domain-containing diguanylate cyclase	43
pBIC_00107		+	Mobile element protein (IS903B)	2
pBIC_00108		+	Hypothetical protein	36
pBIC_00109		+	Putative membrane protein YjcC	12
pBIC_00110		–	Hypothetical protein	4
pBIC_00111		–	Hypothetical protein	16
pBIC_00112		–	Secondary glycine betaine transporter BetU	54
pBIC_00113		–	Mobile element protein (IS1F)	10
pBIC_00114		–	Mobile element protein (IS1F)	18
pBIC_00115		–	Mobile element protein (ISEc11)	53
pBIC_00116		–	Mobile element protein (ISEc11)	23
pBIC_00117		+	Mobile element protein	21
pBIC_00118		+	Mobile element protein	4
pBIC_00119		–	Silver-binding protein	107
pBIC_00120	*cusS*	–	Osmosensitive K+ channel histidine kinase	48
pBIC_00121	*cusR*	–	Copper-sensing two-component system response regulator CusR	139
pBIC_00122	*cusC*	+	Cation efflux system protein CusC precursor	5
pBIC_00123	*cusF*	+	Cation efflux system protein CusF precursor	17
pBIC_00124	*cusB*	+	Cobalt/zinc/cadmium efflux RND transporter membrane fusion protein CzcB family	10
pBIC_00125	*cusA*	+	Cobalt-zinc-cadmium resistance protein CzcAB Cation efflux system protein CusA	7
pBIC_00126	*copG*	+	CopG protein	27
pBIC_00127		+	Lead cadmium zinc and mercury transporting ATPase B Copper-translocating P-type ATPase	14
pBIC_00128		+	hypothetical protein	42
pBIC_00129	*mepM*	–	Cell wall endopeptidase-family M23/M37	49
pBIC_00130		–	Copper-binding protein PcoE	22
pBIC_00131		+	Multicopper oxidase	51
pBIC_00132	*copB*	+	Copper resistance protein B	32
pBIC_00133	*copC*	+	Copper resistance protein CopC	53
pBIC_00134	*copD*	+	Copper resistance protein D	46
pBIC_00135		+	DNA-binding heavy metal response regulator	57
pBIC_00136		+	Heavy metal sensor histidine kinase	33
pBIC_00137		+	Probable copper-binding protein	30
pBIC_00138		–	Hypothetical protein	25
pBIC_00139		–	Mobile element protein	18
pBIC_00140		+	Aquaporin Z	13
pBIC_00141		–	N-acetyltransferase	48
pBIC_00142	*arsR*	+	Arsenical resistance operon repressor	21
pBIC_00143	*arsH*	+	Arsenic resistance protein ArsH	39
pBIC_00144		+	Hypothetical protein	47
pBIC_00145	*arsD*	+	Arsenical resistance operon trans-acting repressor ArsD	9
pBIC_00146		+	Arsenical pump-driving ATPase	4
pBIC_00147		+	Arsenical pump-driving ATPase	4
pBIC_00148		+	Hypothetical protein	12
pBIC_00149		–	Arsenate reductase	91
pBIC_00150		–	Arsenic efflux pump protein	18
pBIC_00151		–	Arsenical pump-driving ATPas	48
pBIC_00152	*arsD*	–	Arsenical resistance operon trans-acting repressor ArsD	77
pBIC_00153	*arsR*	–	Arsenical resistance operon repressor	163
pBIC_00154		+	Hypothetical protein	77
pBIC_00155	*ydeA*	+	YdeA protein	85
pBIC_00156		+	DNA binding protein	47
pBIC_00157		+	Hypothetical protein	37
pBIC_00158		+	Haemolysin expression modulating protein	87
pBIC_00159		+	Hypothetical protein	191
pBIC_00160		+	Hypothetical protein	535
pBIC_00161		+	Hypothetical protein	269
pBIC_00162		+	N-acetyltransferase	333
pBIC_00163		+	Transposase	28
pBIC_00164		–	Hypothetical protein	33
pBIC_00165		–	Mobile element protein	79
pBIC_00166		–	Mobile element protein	28
pBIC_00167		–	Zn-dependent protease	57
pBIC_00168		–	Diguanylate cyclase	170
pBIC_00169		–	Hypothetical protein	210
pBIC_00170	*clp*	–	ATP-dependent Clp protease ATP-binding subunit ClpA	409
pBIC_00171	*hsp*	–	Small HspC2 heat shock protein	795
pBIC_00172		–	DNA binding protein	591
pBIC_00173		–	Transposase	14
pBIC_00174		–	Hypothetical protein	11
pBIC_00175		+	Mobile element protein (ISKpn26)	2
pBIC_00176		+	Mobile element protein (ISKpn26)	15
pBIC_00177		+	Hypothetical protein	7
pBIC_00178		–	Mobile element protein	1
pBIC_00179	*cbbR*	–	RuBisCO operon transcriptional regulator CbbR	2
pBIC_00180		–	Phosphonate dehydrogenase (NAD-dependent phosphite dehydrogenase)	4
pBIC_00181	*phnE*	–	Phosphonate ABC transporter permease protein phnE	5
pBIC_00182		–	Phosphonate ABC transporter phosphate-binding periplasmic component	2
pBIC_00183	*phnC1*	–	Phosphonate ABC transporter ATP-binding protein	3
pBIC_00184		+	Mobile element protein	4
pBIC_00185		–	Mobile element protein (IS26)	6
pBIC_00186		+	TnpA transposase (IS26)	8
pBIC_00187		+	Mobile element protein (partial IS26)	22
pBIC_00188	Δ*bla*_OXA−9_	+	Beta-lactamase class D (partial OXA-9)	6
pBIC_00189	Δ*bla*_OXA−9_	+	Beta-lactamase class D (partial OXA-9)	6
pBIC_00190		+	Mobile element protein (partial Tn3)	241
pBIC_00191	*bla*_TEM−1_	+	Beta-lactamase TEM-1	175
pBIC_00192		–	Mobile element protein (partial IS26)	13
pBIC_00193	*aph(3*′*)-I*	+	Aminoglycoside phosphotransferase	655

*Genes with RPKM values above the RPKM value of IncFII_K_ replicase repA are highlighted in gray*.

Tansformation of pBIC1a into the *E. coli* background only resulted into a slight increase in the MIC for imipenem (0.5 μg/ml compared to 0.25 μg/ml in *E. coli* TOP10 wild-type strain). However, our RNA-seq experiments showed that *bla*_KPC−2_ gene was the most expressed plasmidic gene (Table 1; Figure 3). Two promoters were previously shown to contribute to *bla*_KPC−2_ gene expression (Naas et al., 2012). Although our RNA-sequencing protocol was not designed to precisely map transcription start sites, the high coverage by sequencing reads upstream the p1 promoter sequence strongly suggests promoter p2 as the main promoter for *bla*_KPC−2_ gene transcription (Figure S3). In addition, RNA-seq analysis revealed no clear transcription termination with reads continuously covering more than one kb downstream *bla*_KPC−2_ stop codon (Figure S3). In agreement with this observation, no rho-independent termination site could be predicted by bioinformatic tools (http://rna.igmors.u-psud.fr/toolbox/arnold/). This transcription covered in antisense the transposase gene of IS*Kpn*6. It might therefore act as a repressor for IS*Kpn6* transposition as an antisense RNA, and/or play a role in the regulation of KPC through mRNA stability as observed for other 3′-UTR (Ruiz de los Mozos et al., 2013).

Other resistance genes carried by the plasmid, namely *bla*_TEM−1_ and *aph*(3′)-Ia genes were also highly expressed (Table 1; Figure 3). The high expression of *aph(3*′*)-Ia* gene is in accordance with the high level resistance to kanamycin observed (MIC > 32 μg/ml). The association of *bla*_KPC_ gene with other antibiotic resistance determinants also highly expressed provides a very simple scenario for a carbapenemase to spread as a hitchhiker gene, especially in the absence of carbapenem selection.

Different operons involved in metal resistance were identified on pBIC1a: the *cus (cusCFBA)* and *cop (copABCDR)* operons involved in copper and other cations efflux, and the *ars* operon (*ars*RDABC) in arsenical resistance. Here, only the regulators genes *cusR* and *arsR* were highly transcribed. CusR is part of the two component system CusRS that activates the transcription of *cusCFBA* genes in the presence of Cu/Ag (Xiao et al., 2017). pKPN3-like plasmids carrying the highly similar sil or cus operons are known to confer resistance to high silver concentration in *K. pneumoniae* and in *Enterobacter cloacae* (Finley et al., 2015). On the contrary, ArsR acts as a repressor for the arsenical resistance operon (Ren et al., 2017).

Several mobile elements and insertion sequences (IS) were identified on pBIC1a including one copy of IS*Kpn6*, IS*Kpn7*, IS*Kpn26*-like, IS*Kpn28*, IS*Kpn32*, IS*1*, IS*903*, and IS*Ecl1* (Figure 1). In addition to these elements, several fragments of IS*26* were identified. These IS were not or weakly expressed that is in accordance with the tight regulation of ISs (Nagy and Chandler, 2004). An interesting feature of IS*Kpn31* was the expression of its passenger gene. Whereas, the transposase gene was almost not expressed, its passenger gene (pBIC_0005) belonged to the top expressed genes of the plasmid. This encoded protein is named p019 and has been identified on matrix-assisted laser desorption ionization–time of flight mass spectrometry (MALDI-TOF MS) spectra of *K. pneumoniae* harboring pKpQIL-like plasmids (Lau et al., 2014; Partridge, 2014). The presence of a peptide signal indicates a probable export but this protein does not share homology with protein with known function.

Recently, Buckner et al. studied the expression of two *bla*_KPC_-producing pKpQIL-like plasmids in *K. pneumoniae*, and revealed that 60% of plasmid-encoded genes were expressed. These genes included genes involved in replication, transmission, stability, recombination and toxin-antitoxin systems (Buckner et al., 2018). Comparison of pKpQIL-UK, pKpQIL-D2, and pBIC1a transcripts revealed common expressed genes, including *bla*_KPC_, *bla*_TEM_, *repA, traALMTS, finO, tnp* gene coding for Tn*3* transposase. There are growing evidence for the existence of “core” expressed genes associated to pKpQIL-like plasmids, expressed in both *K. pneumoniae* and *E. coli* genetic backgrounds.

### Imipenem-induced response in *E. coli* (pBIC1a): global changes

After imipenem exposure, we observed a major impact on *E. coli* (pBIC1a) whole transcriptome with 1,563 RNAs being differentially expressed (with adjusted *p*-values ≤ 0.05) among a total of 4,550 (34%). 35% (1,535 over 4,357) of chromosomally-encoded genes and 14% (28 over 193) of pBIC1a genes were affected by imipenem exposure. 765 RNAs were up-regulated and 798 were down-regulated (Table S2). Among differentially expressed genes, 154 RNAs had a Fold-Change (FC) >2 and 329 RNAs had a FC < 0.5. Differential expression of eight selected genes (four up-regulated and four down-regulated genes) was confirmed by RT-qPCR (Figure S4).

### Imipenem-induced response at the plasmid pBIC1a level

Effect of imipenem on all pBIC1a transcripts is indicated in Table S3. After imipenem addition in culture medium, nine predicted CDS of pBIC1a were up-regulated but none with a FC >2 (Table 2). Transcription of copper resistance operon (*copABDRS*/*pcoE*) was induced after imipenem exposure, with FC values < 2. With two oxidation states Cu^+^ and Cu^2+^, copper is a cofactor in redox enzymes that uses dioxygen as a substrate (Rademacher and Masepohl, 2012). Bacteria synthetize various cuproenzymes, which play important roles in cellular processes such as energy transduction, iron mobilization and oxidative stress (Rademacher and Masepohl, 2012). In excess, copper becomes toxic as it interacts with free proteinogenic thiol groups, destabilizes iron–sulfur cofactors, and possibly leads to formation of ROS (Chillappagari et al., 2010; Rademacher and Masepohl, 2012). Activation of the copper resistance operon could act as a defense factor to limit toxicity induced by high copper intracellular concentration, hence ROS production (Bondarczuk and Piotrowska-Seget, 2013). The chromosomally-encoded small RybA (also known as MntS) could be involved in this positive regulation. It was up-regulated after imipenem exposure (FC = 3.5 *p*-value = 4.14e-39). RybA transcripts are stabilized under peroxide stress and members of the CusR regulon are downregulated in a Δ*rybA* mutant, suggesting that RybA could be a positive regulator of genes involved in copper detoxification (Gerstle et al., 2012).

**Table 2:** Differentially expressed genes carried by pBIC1a.

**Id**	**Name**	**Counts (without imipenem)**	**Counts (with imipenem)**	**Fold-Change**	**log2(Fold-Change)**	**Adjusted *p*-value**	**Annotation**
**DOWN-REGULATED GENES**							
pBIC_00006	pBIC_00006	110	77	0.697	−0.522	0.018900674	Transposase (ISKpn31)
pBIC_00007	pBIC_00007	247	110	0.45	−1.152	5E-12	Transposase DDE domain protein
pBIC_00008	pBIC_00008	256	104	0.412	−1.281	1.20E-13	Transposase (ISKpn6)
pBIC_00034	pBIC_00034	4,125	3,052	0.741	−0.432	3.15E-05	Conjugal transfert protein TraG
pBIC_00064	pBIC_00064	3,745	3,173	0.848	−0.238	0.047283777	Lytic Transglycosylase
pBIC_00068	*higA*	4,004	3,293	0.823	−0.28	0.013712564	Antitoxin HigA
pBIC_00069	pBIC_00069	3,600	2,784	0.774	−0.369	0.000846589	Hypothetical protein
pBIC_00097	pBIC_00097	323	204	0.635	−0.655	6.44E-06	Putative transposase
pBIC_00098	pBIC_00098	190	92	0.491	−1.027	5.31E-07	Helix-turn-helix domain protein
pBIC_00108	pBIC_00108	119	50	0.43	−1.218	4.94E-08	Hypothetical protein
pBIC_00109	*yjcC*	450	300	0.669	−0.579	7.65E-06	Putative membrane protein YjcC
pBIC_00168	pBIC_00168	3,282	2,429	0.741	−0.433	0.000196756	Diguanylate cyclase
pBIC_00169	pBIC_00169	1,063	840	0.791	−0.338	0.027066196	Hypothetical protein
pBIC_00170	*clpC*	29,463	22,147	0.753	−0.41	0.000216524	ATP-dependent Clp protease ATP-binding subunit ClpC
pBIC_00171	*hspA*	11,647	7,775	0.669	−0.58	4.35E-07	Heat shock protein
pBIC_00172	pBIC_00172	4,274	2,662	0.624	−0.68	1.51E-08	Helix-turn-helix domain protein
pBIC_00176	pBIC_00176	379	266	0.704	−0.507	0.001793481	Transposase (ISKpn26)
pBIC_00177	pBIC_00177	108	75	0.695	−0.525	0.029045775	Hypothetical protein
pBIC_00186	pBIC_00186	217	165	0.764	−0.388	0.040167906	Transposase (IS26)
**UP-REGULATED GENES**							
pBIC_00010	pBIC_00010	1,807	2,107	1.165	0.22	0.048646696	Hypothetical protein
pBIC_00107	pBIC_00107	48	93	1.911	0.935	0.003155501	Transposase
pBIC_00129	*mepM*	937	1,288	1.374	0.458	1.94E-04	Murein DD-endopeptidase MepM
pBIC_00131	*copA*	2,404	3,018	1.253	0.326	0.003442412	Copper resistance protein A precursor
pBIC_00132	*copB*	725	939	1.291	0.369	0.035026611	Copper resistance protein B precursor
pBIC_00134	*copD*	1,107	1,373	1.239	0.309	0.008349118	Copper resistance protein D
pBIC_00135	*copR*	1,001	1,188	1.185	0.245	0.023543192	Transcriptional activator protein CopR
pBIC_00136	*copS*	1,205	1,491	1.237	0.307	0.009481833	Sensor kinase CopS
pBIC_00137	*pcoE*	336	423	1.255	0.328	0.030486108	Putative copper-binding protein PcoE precursor

*Genes with FC values < 0.5 are highlighted in gray*.

Among the membrane associated regulated genes, a plasmid-encoded murein DD-endopeptidase called *mepM* (pBIC_00129) was up-regulated (FC = 1.37 *p*-value = 1.9e-04). Search for conserved domains using the NCBI Database identified MepM as a member of LytM domain containing proteins, that include numerous autolysins involved in peptidoglycan remodeling (Uehara et al., 2009). Bacteria mutated for such enzymes form longer chains due to a default in cell separation (Visweswaran et al., 2013). On the contrary, overexpression of LysM-associated proteins lead to increased autolysis (Uehara et al., 2009; Visweswaran et al., 2013). This small activation of *mepM* could be an indirect consequence of the interaction of imipenem with its Penicillin Binding Proteins (PBP), reflecting an early activation of bacterial lysis (Uehara et al., 2009), though not observed through time-kill experiments (Figure S1).

The chromosomal copy of *mepM* (also known as *yebA*) of *E. coli* MG1655 was previously shown to be up-regulated under oxidative stress (Seo et al., 2015). Here, we only observed the up-regulation of the plasmid-encoded endopeptidase. The two proteins differed by their size and molecular weight, with plasmid-encoded MepM being a 27.08 kDa protein vs. 49.04 kDa for the chromosomal copy. They shared only 41% of amino-acid identity, suggesting that both enzymes might have different cellular functions.

We specifically looked at the level of *bla*_KPC−2_ gene expression that was not modified in the presence of imipenem (FC = 1.12, *p*-value = 0.14). There is no genetic argument for an inducible expression of *bla*_KPC−2_ and our results were in agreement with a constitutive expression (Naas et al., 2012).

Finally, 19 predicted CDS of pBIC1a were down-regulated with *p*-values < 0.05, and four of them with FC values < 0.5 (Table 2). Six down-regulated genes coded for transposases from various IS. Down-regulation of two helix-turn-helix domain proteins (pBIC_00098 and pBIC_00172) was also observed with *p*-values of 5.31E-07 and 1.51E-08, respectively. Such proteins may act as global regulators for gene expression (Aravind et al., 2005).

### Imipenem-induced response at the chromosomal level

Most of the transcriptomic variations induced by imipenem exposure affected *E. coli* chromosomal genes, with 756 genes (17.4%) being up-regulated and 779 genes (17.9%) down-regulated over 4,357 genes.

**Figure 4:**
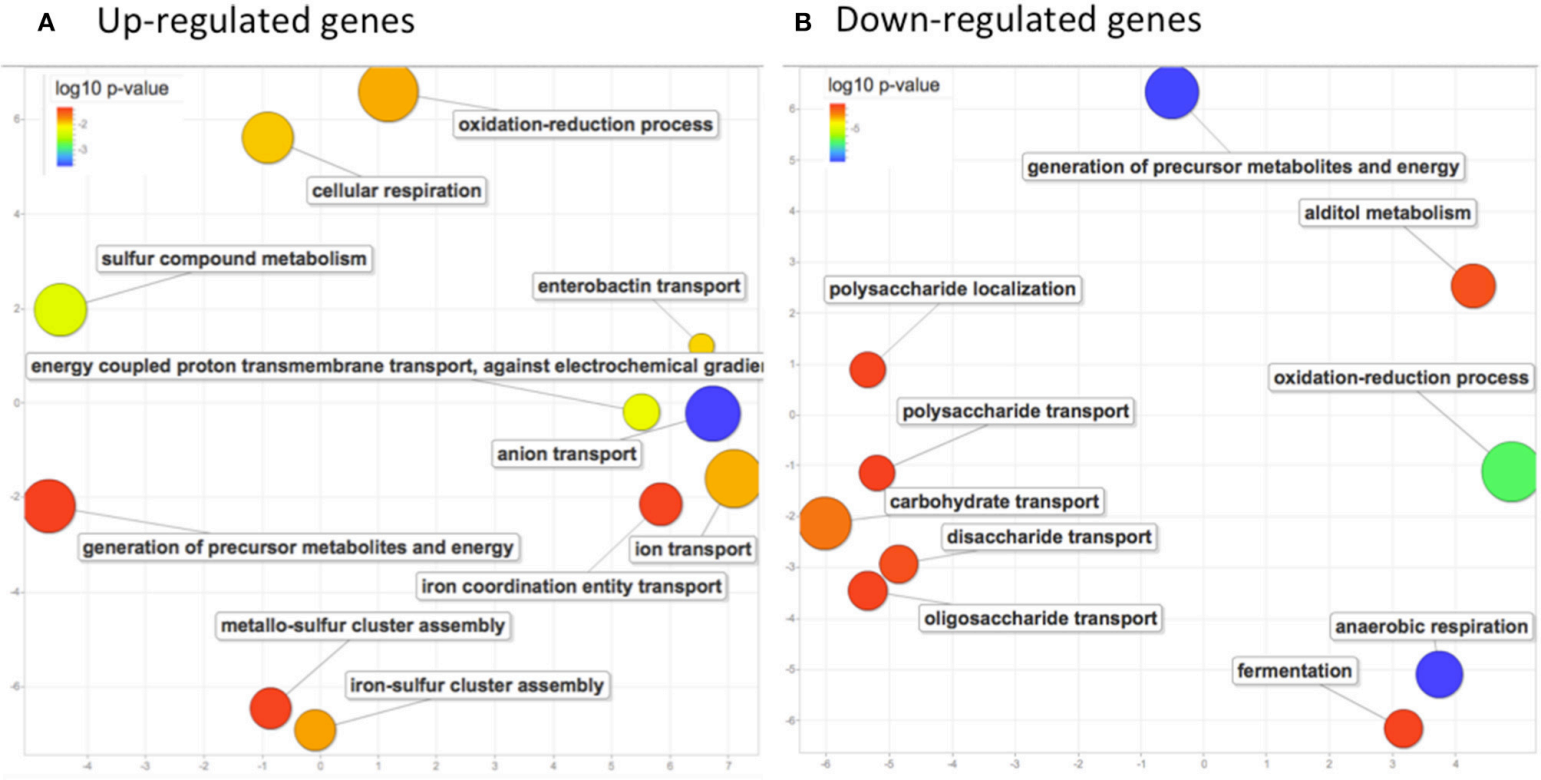
Biological process GO terms enrichment analysis in response to imipenem in *E. coli* (pBIC-1a). Scatterplots showing the non-redundant up **(A)** and down-regulated **(B)** GO terms significantly enriched after imipenem exposure. GO enrichment was determined by PANTHER web application and GO terms significantly enriched with adjusted *p*-values under 0.05 were summarized by using REVIGO. Only chromosomal genes were analyzed. The axes have no intrinsic meaning, similar GO terms remain close together in the plot. The bubble size indicates the frequency of the GO term. **(A)** upregulated genes revealed enrichment in energy production processes (aerobic respiration and electron transport chain), in ion transport and in cluster Fe-S assembly. **(B)** Downregulated genes were mostly associated to anaerobic respiration, generation of precursors of metabolites and energy. Both up and down-regulated genes summarization revealed an enrichment in oxidoreduction process.

To focused on the most significant changes, as a first step, we analyzed global changes on genes with FC values >2 (154 genes) or < 0.5 (325 genes). To identify common metabolic pathways, genes were analyzed for gene ontology (GO) term enrichment using PANTHER web application. Enriched GO terms from up-regulated genes (adjusted *p*-values ranging from 2.23e-4 to 0.053) and from down-regulated genes (adjusted *p*-values ranging from 2.22e-10 to 0.047) were further explored separately on the REVIGO web application to visualize relationships and redundancy among GO terms (Supek et al., 2011; Figure 4). An activation of the aerobic respiration was observed with enrichment in the pathway of electron transport chain (*p*-value = 0.014). Expression of genes involved in cluster Fe-S assembly (*p*-value = 0.016) and in ion transport (*p*-value = 0.015) was also affected. Besides, downregulated genes were mostly associated to anaerobic respiration (*p*-value 2.22e-10) and generation of precursors of metabolites and energy (*p*-value 2.55e-10). Up and down-regulated genes were enriched in oxydo-reduction process (*p*-values 0.015 and 1.82e-07 respectively), suggesting that oxidative stress might have occurred in bacterial cells after imipenem exposure (Figure 4).

### Imipenem induces the activation of several regulons linked to oxidative stress in *E. coli* (pBIC1a)

We searched for genes that could be related to regulons induced by oxidative stress conditions in *E. coli* (Zheng et al., 2001; Blanchard et al., 2007; Seo et al., 2015). Activation of 9 genes that belonged to the SoxRS regulon was observed e.g., the superoxide dismutase *sodA* (FC = 2.1, adjusted *p*-value = 4.6e-20), the fumarate hydratase *fumC* (FC = 2.2, adjusted *p*-value = 8.3e-10), the aconitate hydratase *acnA* (FC = 1.9, adjusted *p*-value = 2.4e-13), the paraquat-inducible membrane protein A *pqiA* (FC = 1.4, adjusted *p*-value = 3.8e-06; Table S4). SoxR is a transcription factor containing [2Fe-2S] clusters, which is the sensor enhancing the expression of SoxS in response to oxidative stress (Lushchak, 2011). Then, SoxS regulates genes implicated in superoxide scavenging, carbon metabolism, DNA repair and xenobiotic efflux (Blanchard et al., 2007). Our transcriptomic data revealed differential gene expression of other regulons linked to oxidative stress response in *E. coli*, such as the OxyR regulon (*grxA, trxC*, …), the IscR regulon for Iron-Sulfur cluster binding and assembly (*iscR, iscA, iscU, iscS, bfd, fdx*…) and the Fur regulon for iron homeostasis and uptake machinery (*fep, fepD, fepG, fecR, efeB, efeU, yncD*…; Table S4).

### Activation of the tricarboxylic acid cycle

Induction of oxidative stress after exposure to β-lactams has been observed in previous studies (Kaldalu et al., 2004; Belenky et al., 2015). Kohanski et al. (2007) and Dwyer et al. (2014) proposed a model explaining how bactericidal antibiotics were able to generate reactive oxygen species (ROS) in bacterial cells in addition to their main mode of action (Kohanski et al., 2007; Dwyer et al., 2014). According to these studies, interactions between β-lactams and Penicillin-Binding Proteins (PBPs) stimulate the oxidation of NADH through the electron transport chain, which is dependent on the tricarboxylic acid (TCA) cycle. Hyperactivation of the electron transport chain stimulates superoxide (O^2−^) formation, which overwhelms superoxide dismutase defenses and leads to the oxidation of iron-sulfur clusters ([4Fe−4S]^2+^) employed by numerous dehydratase enzymes. The release of ferrous (Fe^2+^) irons leads to hydroxyl radicals (OH^*^) *via* the Fenton reaction, which damage DNA, lipids and proteins and therefore contributes to antibiotic-induced cell death.

**Figure 5:**
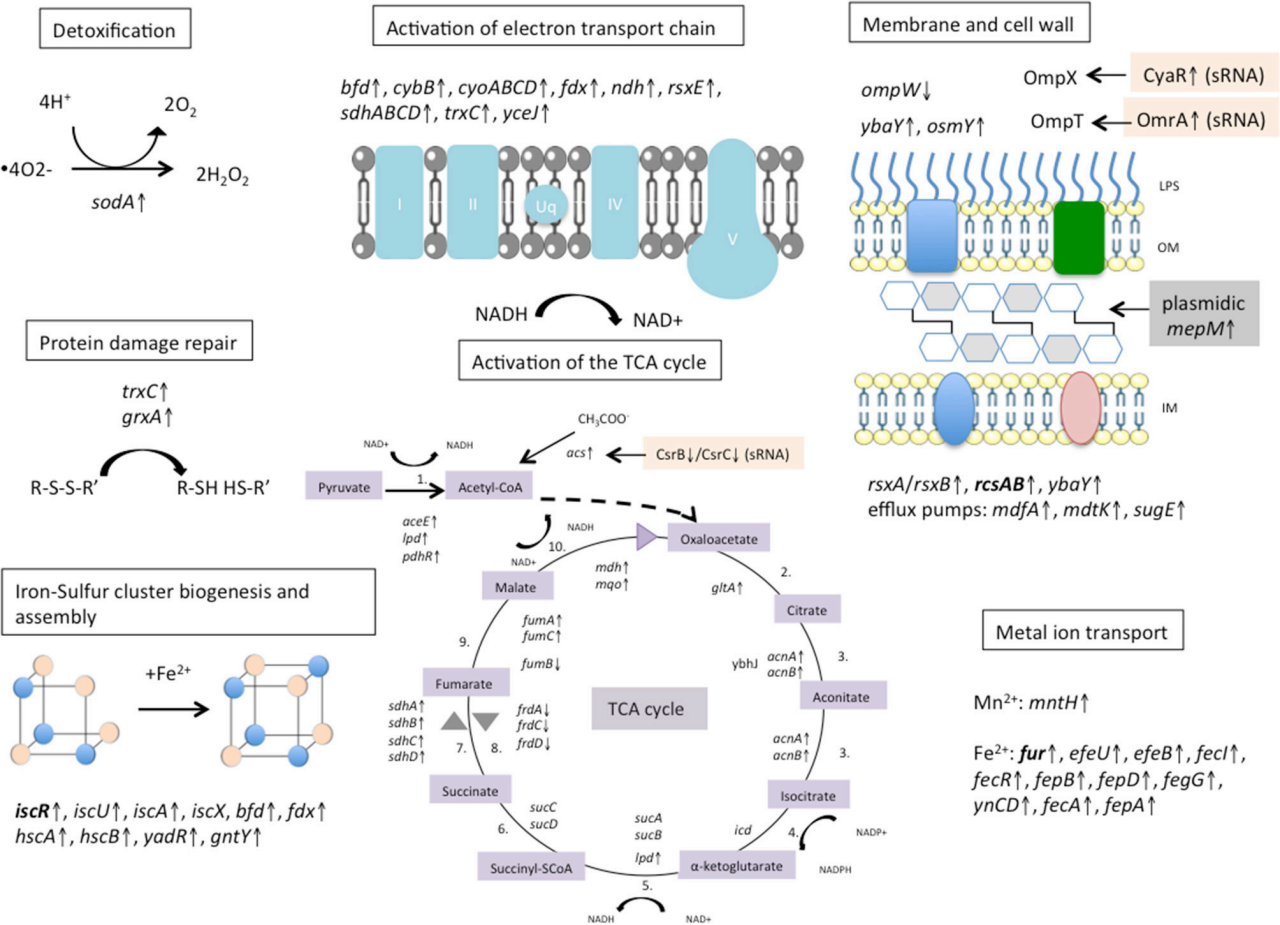
Metabolic response of *E. coli* pBIC-1a to imipenem exposure. Activation of the electron transport chain pathway dependent on the tricarboxylic acid (TCA) cycle was observed, and is thought to generate ROS according to the model proposed by Kohanski et al. and Dwyer et al. (Kohanski et al., 2007; Dwyer et al., 2014). We observed an up-regulation of most of the TCA cycle enzymes, of many genes involved in Iron–Sulfur cluster biogenesis and assembly, and of several metal ions permeases that are assumed to fuel the Fenton reaction. Detoxification process occured to limit protein damages and ROS accumulation with up-regulation of superoxide dismutase *sodA*. Several genes involved in bacterial cell wall homeostasis were disturbed. Activation of the plasmidic murein endopeptidase *mepM* might modify bacterial peptidoglycan and reflect an early activation of bacterial lysis. Transcriptional regulators are written in bold. ↑ means up-regulated and ↓ means down-regulated. LPS, lipopolysaccharide; OM, outer membrane; IM, inner membrane. Reactions of the TCA cycle: 1. Pyruvate dehydrogenase, 2. Citrate synthase, 3. Aconitase, 4. Isocitrate dehydrogenase, 5. α-ketoglutarate dehydrogenase, 6. SuccinylCoA thiokinase, 7.Succinate dehydrogenase, 8. Fumarate reductase 9. Fumarase, 10. Malate dehydrogenase.

Activation of the TCA cycle and new iron-sulfur cluster synthesis have been shown to play critical roles in the initiation of the oxidative stress response after antibiotic exposure (Kohanski et al., 2010). Interestingly, our transcriptomic data revealed an overexpression of almost all TCA enzymes (Table S2; Figure 5).

A critical parameter to suppress Fenton reaction is the scavenging of H_2_O_2_ generated in bacterial cells by *sodA*. This can occur through the activation of peroxidases like KatG, KatE, AhpC, AhpF, or the protein complex Hcp-Hcr being a putative hydroxylamine reductase or a peroxidase (Wolfe et al., 2002; Almeida et al., 2006). Here, only the two copies of *ahpF* were slightly up-regulated (with FC values < 2). Of note, *katG* and *hcp-hcr* were even down-regulated. Our results suggest that no H_2_O_2_ detoxification was highlighted by RNA-Seq after 10 min of imipenem exposure. Microarray data collected by Dwyer et al. also observed down-regulation of *katG* and *hcp-hcr* in *E. coli* MG1655 exposed to ampicillin (Dwyer et al., 2014). Nevertheless, in this study, overexpression of *E. coli katG* and *ahpCF* partially protected the bacteria from β-lactams lethality, confirming the protective role these peroxidases.

### Genes related to Fe–S clusters biogenesis and iron recruiting

In addition, we observed up-regulation of numerous Fe-S cluster binding proteins and Fe-S cluster assembly proteins (*iscS, iscU, iscA, yadR*, and *gntY*) indicating an activation of the Fe-S cluster biogenesis. Consistent with this, genes encoding proteins related to iron uptake and storage were regulated following imipenem exposure. Several iron permeases were up-regulated such as *efeU* (ferrous iron permease) (validated by RT-qPCR), *efeB* (exogenous heme iron acquisition), *fecI* (minor sigma factor that initiates transcription of ferric citrate transport genes), *fecR, fepB, fepD, fegG* (ferric enterobactin ABC transporter complex), and *yncD* (outer membrane protein involved in iron transport). In parallel, *bfd* gene encoding iron storage protein bacterioferritin-associated ferredoxin was up-regulated but not its partner coding the bacterioferritin Bfr. Bfd and Bfr can store iron as ferric iron to protect cells against ROS resulting from ferrous iron overload (Andrews et al., 2003).

The uptaken Fe^2+^ could also be reintegrated into Fe-S clusters to stabilize dehydratases and therefore be a metabolic feedback to counteract antibiotic toxicity (Py and Barras, 2010). YtfE is a protein known to recruit and integrate Fe^2+^ to repair Fe-S cluster damaged proteins (Py and Barras, 2010). Here, *ytfE* was not induced in our conditions (FC = 0.816, *p*-value = 0.16), suggesting that no suppression of Fenton reaction occurred through *ytfE*. Therefore, Fe^2+^ uptake could directly fuel Fenton reaction to produce the highly reactive HO^*^ and contribute to maintain oxidative stress.

### Genes involved in cell wall

Imipenem is an antibiotic targeting bacterial cell wall biogenesis. Therefore, we looked at cell envelope components that could be implicated in reducing entry of antibiotic or in resistance to osmotic stress.

Among the osmotic stress-inducible genes, *ybaY, bdm*, and *osmY* were up-regulated. YbaY is a lipoprotein associated to various stress responses like the oxidative stress response (Cheung et al., 2003), the RpoS-induced stress (Weber et al., 2005), and the osmotic stress response (Asakura and Kobayashi, 2009). Bdm is involved in flagellar biosynthesis, and its overexpression, regulated by the RcsB transcriptional activator, enhances biofilm formation (Sim et al., 2010; Kim et al., 2015). Here, RcsA/RcsB regulators that belong to the multicomponent RcsF/RcsC/RcsD/RcsA-RcsB phosphorelay system were indeed up-regulated upon imipenem addition (Gottesman and Stout, 1991; Majdalani and Gottesman, 2005). Any perturbation of the peptidoglycan activates the Rcs phosphorelay, and RcsB activity enables bacteria to survive in the presence of the antibiotics (Laubacher and Ades, 2008), possibly due to a modification of bacterial cell wall through synthesis of the colanic acid capsular exopolysaccharide. Modification of expression of other genes belonging to the Rcs regulon and involved in flagellar biosynthesis was observed, like the downregulation of *flhC*/*flhD*.

Few genes associated to membrane components were down-regulated, like *ompW* coding for an outer-membrane porin. Expression of *ompW* was validated by RT-qPCR (Figure S4). OmpW has been implicated in the adaptation to stresses in various species, but its biological function is not yet fully characterized. *ompW* expression was decreased in *E. coli* in response to oxidative stress by an unknown mechanism (Blanchard et al., 2007). Recently, Xia et al. revealed a role in the carbon and energy metabolism in *E. coli* (Xiao et al., 2016). Expression of *ompW* is modulated by various regulators including RybB, a small RNA dependent on the alternative sigma factor σ^E^, and the transcriptional regulator FNR (Fumarate and Nitrate Reduction) particularly important during anaerobic transition (Xiao et al., 2016). Here, both *rybB* and *fnr* were overexpressed after imipenem addition (FC = 1.546; *p*-value = 0.023 and FC = 2.38; *p*-value = 1.1e-46 respectively) and thus probably involved in *ompW* regulation.

In parallel to *ompW* down-regulation, several efflux pumps were up-regulated, *mdfA* (also known as *cmr*) *mdtK* and *sugE*, with FC values < 2. These proteins are part of multi-drugs efflux pumps and could help bacteria to get rid of carbapenems and/or oxidative compounds.

### Small RNAs

Bacterial small RNAs are major actors for bacterial adaptation to various stresses and some of them are involved in regulatory circuits controlling antibiotic resistance (Felden and Cattoir, 2018). For instance, sRNA can control antimicrobial intracellular concentration by regulating the expression of several outer membrane proteins and efflux pumps. They can also regulate the expression of bacterial component like the LPS or enzymes involved in cell wall biosynthesis (Felden and Cattoir, 2018).

Following imipenem exposure, 14 sRNAs were differentially expressed (Table S5): 11 were up-regulated and 3 were down-regulated.

IsrB, also known as AzuC, was the most induced sRNA (FC = 10.334; *p*-value = 4.26e-80). No target has been clearly identified to date, but hydrogen peroxide treatment could also induce AzuC expression 4-fold in a previous study in *E. coli*, suggesting a role in oxidative stress response (Hemm et al., 2010).

*cyaR* (also known as *ryeE*) (FC = 1.93; *p*-values = 1.33e-12) and *omrA* (also known as *rygA*) (FC = 1.88; *p*-value = 6.3e-5) are known to decrease the expression of outer membrane proteins OmpX and OmpT, respectively (Vogel and Papenfort, 2006; Rau et al., 2015). However, it is not clear whether this down-regulation occurs at the translational level or through the decay of the mRNA targets (Vogel and Papenfort, 2006). Here, we could not observe a down-expression of *ompX* and *ompT* suggesting that base-pairing of both sRNAs with their targets does not affect RNA stability but would essentially interfere with the translation of the outer membrane proteins. OmpT belongs to aspartyl proteases, involved in virulence by protecting cells from cationic antimicrobial peptides, activation of the anticoagulation pathway, and inflammatory responses (Hwang et al., 2007). OmpX mediates adhesion to eukaryotic cells and negatively regulates porins involved in β-lactam uptake like OmpK35 and OmpK36 in *Enterobacter aerogenes* (Dupont et al., 2004; Kim et al., 2010). Its expression also increases under high osmolarity conditions (Dupont et al., 2004). Inhibition of *ompX* translation through interaction with CyaR might indirectly increase carbapenem influx in bacterial cells.

Another target of OmrA is the transcriptional regulator CsgD that controls biofilm formation (Mika and Hengge, 2014). In biofilms, bacteria survive as persister cells and become more tolerant to antibiotics. By downregulating *csgD* and therefore biofilm formation, the expression of OmrA could increase susceptiblity to antimicrobial agents.

*csrB* and *csrC* were less expressed in the presence of imipenem. These two sRNA regulate the carbon storage regulator *csrA*, which activates the expression of several metabolic enzymes involved in energy conversion (Weilbacher et al., 2003). One of the targeted enzymes is the Acetyl CoA synthase (FC = 1.34, *p*-value = 0.006), which when induced may contribute to fuel the TCA cycle by a secondary pathway.

RybA (also known as MntS) was also an up-regulated small RNA during imipenem exposure. As previously mentioned, RybA transcripts could be implicated in the up-regulation of the plasmid copper resistance operon (see section Imipenem-induced response at the plasmid pBIC1a level).

### Conclusions

Taken together, our work showed that imipenem induced an oxidative stress response in *E. coli* TOP10(pBIC1a) and perturbed several regulatory networks and cellular compounds (Figure 5). In particular, treatment with high concentrations resulted in the activation of the TCA cycle, the electron transport chain pathway and iron metabolism. Few enzymes were induced supposedly to limit the damages caused by oxidative stress, such as detoxification by superoxide dismutase SodA, and protein damage repair by thioredoxins and glutaredoxins (Figure 5). Whereas, the effect on chromosomal genes was highly significant, the impact of imipenem exposure on the transcription of plasmid genes was more limited. However, we could identify few differentially expressed genes that could be involved in resistance to these stressful conditions.

β-lactams are also known to trigger a SOS response in Gram-negative bacilli (Michel, 2005) but activation of the SOS response was not evidenced here. SOS response can be induced when bacteria are exposed to subinhibitory concentrations of antibiotics. In this study, high doses of imipenem were used instead to reproduce the effect of antibiotic concentrations reached in human serum. Another explanation might be that SOS response depends on the PBPs targeted by the β-lactam (Miller, 2004). After exposure to ampicillin, PBP3 inhibition causes filamentation and is known to stimulate the DpiAB two-component system, which activates the SOS response (Miller, 2004). At the opposite, imipenem preferentially targets PBP2 (Davies et al., 2008). By targeting preferentially some PBP, β-lactams seem to induce different stress responses.

Dissemination of KPC-encoding plasmids in Gram-negative bacteria poses a serious threat to medical treatment and patient management. To better understand the plasmid biology of a prototypical successful IncFII_K_-IncFIB *bla*_KPC−2_-carrying plasmid, a transcriptomic analysis was performed in *E. coli* TOP10, which constituted a well-characterized and annotated model. The choice of the genetic background is an important matter and has probably an impact on the expression of plasmid genes. Given the strong association between IncFII_K_ plasmids and *K. pneumoniae* CG258, further studies using *K. pneumoniae* background are needed to understand the interaction between successful plasmids and successful clones. Here, genes involved in antibiotic resistance and basic plasmid function such as replication were highly expressed in broth culture. Carbapenem exposure did not influence the level of transcription of antimicrobial resistance genes. However, despite high transcription of *bla*_KPC−2_ gene, we observed a major impact of imipenem on chromosomal genes and identified adaptive responses of *E. coli* to imipenem-induced oxidative stress.

## Methods

### Bacterial transformation

*K. pneumoniae* BIC-1 is a KPC-producing clinical strain isolated at Bicêtre Hospital in 2009 (Naas et al., 2010). This strain has been entirely sequenced using PacBio and Illumina sequencing (Jousset et al., 2018). The natural IncFII_K_-IncFIB plasmid carrying *bla*_KPC−2_ gene, named pBIC1a was extracted from the BIC-1 strain by using the Kieser extraction method and subsequently analyzed by electrophoresis on a 0.7% agarose gel (Kieser, 1984). Plasmid pBIC1a was then introduced by electroporation into electrocompetent *E. coli* TOP10 (Invitrogen, Eragny, France) using a Gene Pulser II (Bio-Rad Laboratories. Marnes-la-Coquette, France). The transformant *E. coli* TOP10-pBIC1a was selected on plates supplemented with ticarcilline 50 mg/L (Sigma-Aldrich). The presence of pBIC1a was verified by PCR and by plasmid extraction followed by electrophoresis on a 0.7% agarose gel.

### pBIC1a sequence analysis

Annotation of pBIC1a was performed using PROKKA (Seemann, 2014). Mobile element proteins and insertion sequences were annotated with ISFinder (https://www-is.biotoul.fr/index.php; Siguier et al., 2006). Sequences of plasmids pKPN3 (NC_009649.1), pKpQIL (GU595196.1), pKpQIL-IT (NC_019155.1), pITO6-C07 (LT009688.1), pBK32179 (JX430448) and pGMI16-005-01 (NZ_CP028181.1) were used for genomic comparison. Plasmid alignment was performed using BRIG (Alikhan et al., 2011) and sequence similarity was studied using BLAST software (https://blast.ncbi.nlm.nih.gov/Blast.cgi). Allelic variant of the replicase genes were analyzed by replicon sequence typing using Plasmid Finder (https://cge.cbs.dtu.dk/services/PlasmidFinder/; Carattoli et al., 2014).

### Time-kill experiments

Time-kill experiments were performed to evaluate bacterial cell death after 10 min of imipenem exposure. Briefly, 10 ml of a log-phase culture (OD_600_ at 0.5) of *E. coli* TOP10-pBIC1a was divided in two tubes of 5 ml. A final concentration of imipenem at 5 μg/ml was added in one tube. After 10 min, 10-fold serial dilutions were performed and 100 μl of diluted cultures were plated on agar plates without antibiotic. All these steps were performed in triplicates. The next day, colony-forming units (CFU) were counted.

### RNA isolation and purification

An overnight culture of *E. coli* TOP10-pBIC1a was diluted 100-fold in fresh BHI broth and allowed to grow to an OD_600_ of 0.5. The culture was then split and imipenem (Imipenem-Cilastatin, Mylan, USA) was added to one of the cultures at a final concentration of 5 μg/ml. Ten min later, 2 ml of each culture were taken and mixed with RNA protect (Qiagen, Courtaboeuf, France) according to the manufacturer's recommendations. Cells were immediately pelleted by a 10 min centrifugation at 5,000 g. Biological triplicates were performed.

RNA was extracted using GeneJet RNA extraction kit (ThermoScientific, ThermoFisher Scientific, Villebon sur Yvette. France). DNAse treatment was performed with the dsDNAse kit (ThermoScientific) according to the manufacturer's protocol. RNA concentration was measured using Qubit RNA BR assay kit (Invitrogen. ThermoFisher Scientific), and RNA quality was evaluated using the Agilent 2100 Bioanalyzer RNA 6000 Nano Kit (Agilent Technologies, Santa Clara, CA). RIN >9 were obtained for all the RNA samples. Verification of complete removal of any contaminating DNA was performed via PCR amplification of *recA* housekeeping gene. A total of 2.5 μg of total RNA was treated with the Ribo-Zero rRNA Removal kit according to the manufacturer's instructions (Illumina, CA, USA) to remove 16S and 23S rRNAs. rRNA removal efficiency was then analyzed via the Bioanalyzer RNA 6000 Nano Kit. Ribodepleted RNA concentration was measured using Qubit™ RNA HS assay kit (Invitrogen).

### RNA library and sequencing

cDNA libraries were prepared using the NEBNext® Ultra Directional RNA Library Prep Kit for Illumina protocol (New England Biolabs, MA, USA). The quality of the cDNA was validated using the Agilent 2100 Bioanalyzer DNA1000 kit (Agilent Technologies) and quantity was determined with the Qubit dsDNA BR Assay (ThermoFisher Scientific). Sequencing of the library was performed with an Illumina HiSeq 2500 in single-read mode with 50 cycles.

### RT-qPCR

Eight up and down-regulated genes were selected and expression levels were analyzed by quantitative RT-PCR to validate the RNA-Seq data. The primers used are listed in Table S6. An input of 100 ng of total RNA, with no contaminating DNA was used. One-step RT–qPCR was performed using One-step RT-PCR kit (Qiagen) according to the manufacturer's protocol, and the reactions were carried out in 96-well plates with CFX96 Real-time PCR System (BioRad, USA). All qRT-PCRs included an initial denaturation step of 30 s at 95°C, 35 cycles of denaturation (95°C/1s), annealing (52°C/5s), and extension (72°C/7s). Expression of each gene was normalized using *rpoB, idnT* and *yiaJ* genes as previously described (Zhou et al., 2011). Expression of these genes was not influenced by the presence of imipenem according to the RNA-Seq data. All reactions were carried out in triplicates. The relative abundance of gene transcripts among the imipenem treated group was calculated using the 2^−ΔΔ*Ct*^ method (Livak and Schmittgen, 2001).

### RNA-seq data analysis and differential expression analysis

Reads generated from strand-specific RNA-seq experiments were aligned to the genome of *E. coli* TOP10 genome (CP000948.1) and to that of pBIC1a sequence (CP022574.1) by using the software Bowtie (version 0.12.7) (Langmead et al., 2009). Reads that mapped in more than four different positions on the genome were discarded i.e., reads corresponding to rRNA. RNA-seq data were analyzed as described (Rosinski-Chupin et al., 2015) by using Rsamtools (version 1.26.2), GenomicAlignments (version 1.10.1). GenomicFeatures (version 1.26.4) and DESeq2 (version 1.14.1) and SARTools (Varet et al., 2016) in R 3.3.1. Only adjusted *p*-values were used and were obtained using the Benjamini–Hochberg correction for false discovery rate (Benjamini and Hochberg, 1995). Read count data were visually assessed using the Artemis genome viewer (Carver et al., 2008). The coverage (expressed as the average density of reads over 500 nucleotide fragments) was computed on each strand of pBICa sequence and visualized by using a heatmap representation (IGV software) (Robinson et al., 2011). The gene expression values were quantified in terms of reads per million (RPKM) defined as the total number of reads mapping to the feature divided by feature length (in kbp) normalized by the total number of reads (in millions) (Mortazavi et al., 2008).

The significantly up- and downregulated genes were analyzed for GO term enrichment separately with PANTHER (http://pantherdb.org/webservices/go/overrep.jsp; Mi et al., 2017) using batch upload, and the significantly enriched terms further explored on the REVIGO web application (http://revigo.irb.hr/) to identify and visualize relationships among the GO terms (Supek et al., 2011). The default settings were used on REVIGO with a list of results of medium size, and *E. coli* GO terms database used as reference.

The functions of genes and gene regulons were annotated using the following web databases: EcoCyc (http://biocyc.org/ECOLI/organism-summary; Keseler et al., 2017) and RegulonDB v8.2 (http://regulondb.ccg.unam.mx/index.jsp). Regulation for genes in the SoxRS, OxyR, IscR and Fur regulons were identified as annotated in RegulonDB v8.2 (Gama-Castro et al., 2016).

## Accession numbers

Plasmid pBIC1a has been deposited in NCBI database under accession number CP022574.1, and RNA-Seq data in Array Express under accession number E-MTAB-7190.

## Author contributions

AJ was in charge of the study design, library preparations, RNA-seq experiments, data analysis, and writing of the manuscript. IRC performed statistical analysis, data analysis and the proofreading of the manuscript. JT realized qRT-PCR and Time kill experiments. RB worked on study design, data analysis and the proofreading of the manuscript. PG and TN were in charge of the study design, and the proofreading of the manuscript.

### Conflict of interest statement

The authors declare that the research was conducted in the absence of any commercial or financial relationships that could be construed as a potential conflict of interest.

## Supplementary material

Figure S1 Time-kill analysis after imipenem exposure. Bacteria were diluted using 10-fold serial dilutions and Colony-Forming Units (CFUs) were counted before (T0) and 10 min after imipenem addition (T10). The percentage of surviving bacteria is represented. The experiments were performed in triplicates.

Figure S2 Differential expression of *E. coli* (pBIC1a) genes in presence of imipenem (imi). Condition without antibiotic was used as control (Ct). 479 significantly differentially expressed genes were found (*p*-value < 0.05). **(A)** Plots of the principal component analysis (PCA). All experiments were performed with biological triplicates. **(B)** Volcano plot with 325 genes with diminished expression and 154 genes with increased expression in presence of imipenem are specified.

Figure S3 Transcriptional landscape of Tn*4401a* bracketing *bla*_KPC−2_ carbapenemase gene. **(A)** Strand-oriented read mapping of Tn*4401a* using Artemis software (Carver et al., 2008). No clear transcription termination of *bla*_KPC−2_ transcripts was observed, and this transcription covered in antisense the transposase gene of IS*Kpn*6. CDS and their orientations are indicated by colored arrows in the lower part. The six reading frames are indicated with vertical bars representing stop codon. Green reads represent multiple identical reads merged and black reads represent unique read mapped. **(B)** Mapping of the reads indicates promoter p2 as the main promoter. Promoters are indicated by black boxes and named according to the literature (Girlich et al., 2017). Inverted repeat right (IRR) of IS*Kpn7* is indicated by horizontal black arrow.

Figure S4 RT-qPCR validation of eight representative genes with significantly differential expression after imipenem-induced stress. RT-qPCR data are represented as mean ± SD from three biological replicates.

Table S1 Alignment of raw data on E. coli TOP10(pBIC1a) sequence.

Table S2 Differentially expressed genes after imipenem addition in broth culture (5μg/ml).

Table S3 Effet of imipenem on pBIC1a genes.

Table S4 SoxRS/OxyRS/IscR/Fur-regulated gene expression changes.

Table S5 Differentially expressed regulatory RNAs.

Table S6 Primers used for RT-PCR.
