# Supplementary figures and images for: Transcriptional Landscape of a blaKPC-2 Plasmid and Response to Imipenem Exposure in Escherichia coli TOP10
Source: Front Microbiol. 2018 Dec 3;9:2929. doi: 10.3389/fmicb.2018.02929 (PMC6286996; doi:10.3389/fmicb.2018.02929)

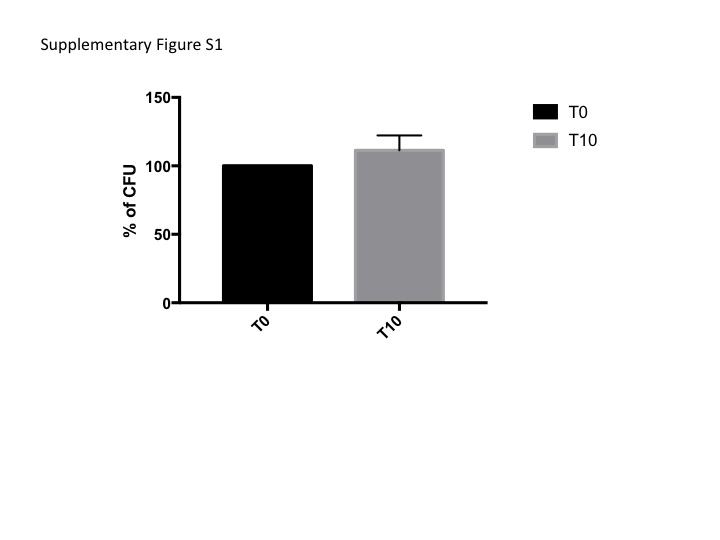

Supplement: Figure S1 — Time-kill analysis after imipenem exposure. Bacteria were diluted using 10-fold serial dilutions and Colony-Forming Units (CFUs) were counted before (T0) and 10 min after imipenem addition (T10). The percentage of surviving bacteria is represented. The experiments were performed in triplicates. [file Image_1.tiff]

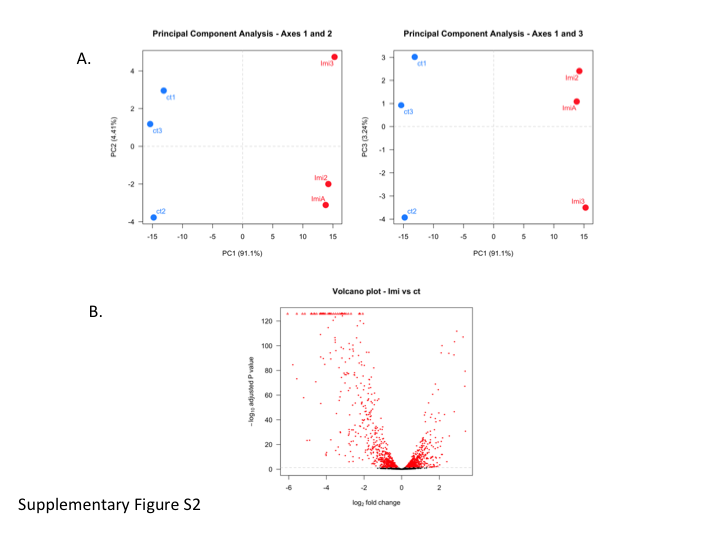

Supplement: Figure S2 — Differential expression of E. coli (pBIC1a) genes in presence of imipenem (imi). Condition without antibiotic was used as control (Ct). 479 significantly differentially expressed genes were found (p-value < 0.05). (A) Plots of the principal component analysis (PCA). All experiments were performed with biological triplicates. (B) Volcano plot with 325 genes with diminished expression and 154 genes with increased expression in presence of imipenem are specified. [file Image_2.tiff]

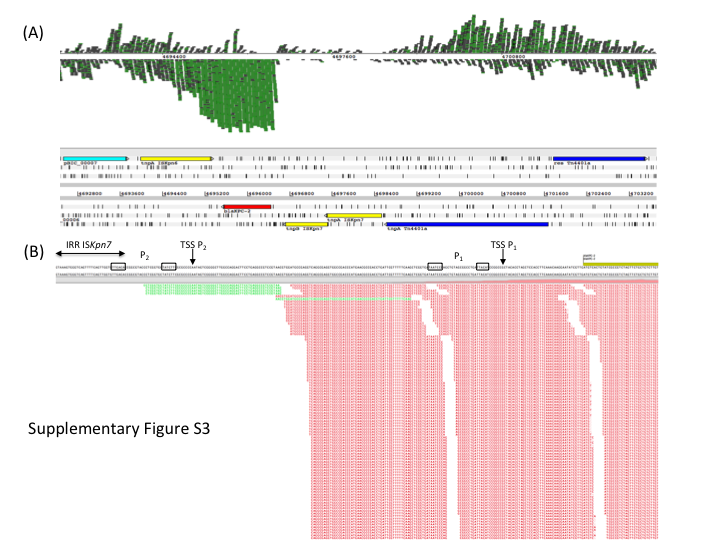

Supplement: Figure S3 — Transcriptional landscape of Tn4401a bracketing blaKPC−2 carbapenemase gene. (A) Strand-oriented read mapping of Tn4401a using Artemis software (Carver et al., 2008). No clear transcription termination of blaKPC−2 transcripts was observed, and this transcription covered in antisense the transposase gene of ISKpn6. CDS and their orientations are indicated by colored arrows in the lower part. The six reading frames are indicated with vertical bars representing stop codon. Green reads represent multiple identical reads merged and black reads represent unique read mapped. (B) Mapping of the reads indicates promoter p2 as the main promoter. Promoters are indicated by black boxes and named according to the literature (Girlich et al., 2017). Inverted repeat right (IRR) of ISKpn7 is indicated by horizontal black arrow. [file Image_3.tiff]

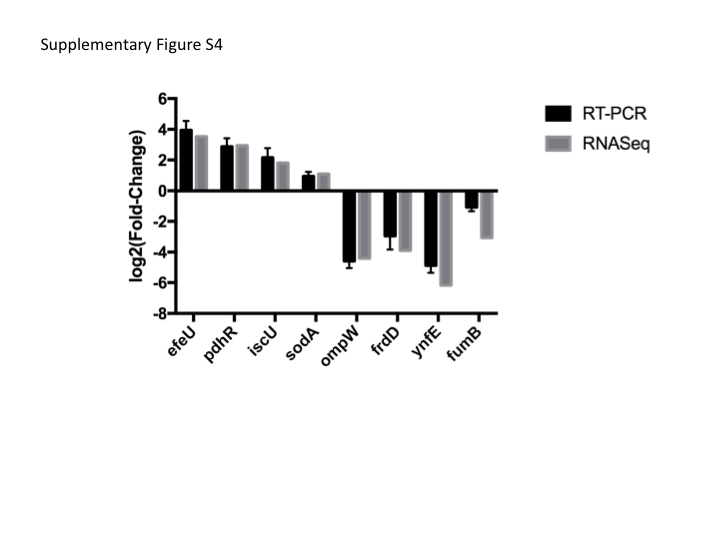

Supplement: Figure S4 — RT-qPCR validation of eight representative genes with significantly differential expression after imipenem-induced stress. RT-qPCR data are represented as mean ± SD from three biological replicates. [file Image_4.tiff]
